# Symptoms of depression in middle-aged and older adults with schizophrenia in Guangzhou, China: a cross-sectional study

**DOI:** 10.3389/fpsyt.2025.1653980

**Published:** 2025-10-01

**Authors:** Junrong Ye, Jialan Wu, Lingli Lei, Dandan Zheng, Yanheng Wei, Dingjie Liu, Xueyu Zheng, Jinrong Li, Na Ma, Yuhan Zhang, Jiao Chen, Jianxiong Guo, Aixiang Xiao

**Affiliations:** ^1^ The Affiliated Brain Hospital, Guangzhou Medical University, Guangzhou, China; ^2^ Key Laboratory of Neurogenetics and Channelopathies of Guangdong Province and the Ministry of Education of China, Guangzhou Medical University, Guangzhou, China; ^3^ Department of Social Psychiatry, The Affiliated Brain Hospital, Guangzhou Medical University, Guangzhou, China; ^4^ Guangdong Engineering Technology Research Center for Translational Medicine of Mental Disorders, Guangzhou, China; ^5^ Department of Nursing, Dongguan Seventh People’s Hospital, Dongguan, China; ^6^ Department of Nursing, The Fifth People's Hospital of Nanning, Nanning, China; ^7^ School of Nursing, Guangzhou Medical University, Guangzhou, China

**Keywords:** middle-aged and older adult, schizophrenia, symptoms of depression, mental health, quality of life

## Abstract

**Objective:**

This study aimed to explore the prevalence and risk factors of symptoms of depression in middle-aged and older adults with schizophrenia.

**Methods:**

In this cross-sectional study, 116 participants were recruited from a tertiary psychiatric hospital in Guangzhou, China. The sample consisted of adults aged 40 years and over. Based on their scores on the Geriatric Depression Scale, participants with schizophrenia were categorized into two groups: those with depressive symptoms (scores greater than 10 points) and those without depressive symptoms (scores of 10 points or lower). This study compared the differences in the sociodemographic variables (such as gender, age, education level, marital status, and BMI) and the clinical characteristics (e.g., suicide risk, anxiety level, severity of mental symptoms, insight into illness and treatment attitude, cognitive function, activities of daily living, quality of life, and social support) between two groups. Spearman’s correlation analysis and logistic regression analysis were employed to explore the relevant factors contributing to depression in middle-aged and older adults with schizophrenia.

**Results:**

The participants had an average age of 63.28 ± 7.87 years, and 88 of the participants (75.9%) are women. Among them, 34 participants (29.31%) exhibited symptoms of depression. Statistically significant differences were observed between the two groups in the affect and resistance sub-dimensions of the Brief Psychiatric Rating Scale (BPRS) and the total scores of the Generalized Anxiety Disorder (GAD) assessment, the Insight and Treatment Attitudes Questionnaire (ITAQ), the Nurses’ Global Assessment of Suicide Risk (NGASR), and the Schizophrenia Quality of Life Scale (SQLS). Furthermore, logistic regression indicated that the resistance sub-dimension of the BPRS (OR = 0.790, 95%CI = 0.648–0.963) and the SQLS (OR = 1.115, 95%CI = 1.055–1.178) and GAD scores (OR = 1.205, 95%CI = 1.029–1.412) are predictive factors for symptoms of depression in individuals with schizophrenia.

**Conclusion:**

In middle-aged and older adults with schizophrenia, affective symptoms and quality of life are associated with symptoms of depression.

## Introduction

Symptoms of depression are common among patients with schizophrenia (SP) (1). Previous studies have indicated that the prevalence of depressive symptoms in this population range from 7% to 75% (2, 3). A survey conducted by Bartels and Drake (4) estimated the prevalence of depression at 20%–70%, while Siris and Bench (5) found the prevalence to range from 7% to 75% in 2000. Wolfram et al. (3) carried out a longitudinal study in Germany over a period of 134 months and found that 60% of the participants (64/107) exhibited at least one depressive symptom at the initial assessment. After 5 years of treatment, the prevalence of depressive symptoms decreased to 40% and subsequently stabilized. Depression occurring at the onset of SP might correlate with a more positive prognosis. However, other studies have shown that depressive symptoms in individuals with chronic SP can lead to disability, increased suicide risk, disease relapse, and readmission (6–8). It has been suggested that depressive symptoms might increase the disease burden for patients with SP. Li et al. (9) demonstrated that nearly half of first-episode or untreated individuals with SP in China were complicated with major depressive episode (MDE). In addition, there may be a significant number of individuals with SP who experience sub-syndromal depressive symptoms (SDS), leading to a broader understanding of the impact of depression in this demographic.

Although researchers have been studying depressive symptoms in middle-aged and older adults with SP for a long time, this is still a topic of debate among scholars. Some believe that depressive symptoms are a core feature of SP (10, 11), while others argue that depressive symptoms are a common complication of mental disorder (12). Many studies have investigated the factors related to these depressive symptoms. Patients with SP might experience different prevalence rates of depression depending on their age. Over 60% of middle-aged and older adults with SP have been reported as experiencing depressive symptoms (7). Comparatively, the occurrence of depressive symptoms in this group is significantly higher than that in age- and gender-matched control groups (7, 13). Among middle-aged and older individuals with SP, depressive symptoms are linked to various negative outcomes, including disability, reduced quality of life (QOL), increased health service utilization, greater severity of positive symptom, demoralization, poorer physical health, low motivation, and suicidal ideation (6–8, 14). Zisook et al. (8) found that the severity of depressive symptoms in middle-aged and older adults with SP was correlated with positive symptoms, but not with gender, age, negative symptoms, extrapyramidal symptoms, or the antipsychotic drug dosage. Furthermore, some studies have suggested that the severity of depression in this population is related to the decline in daily functioning, physical abilities, and overall QOL. However, these depressive symptoms do not appear to be associated with the demographic characteristics, extrapyramidal symptoms, tardive dyskinesia, neurocognitive performance, or the number of physical diseases (7). Diwan et al. (13) also identified several factors associated with depressive symptoms in middle-aged and older adults with SP, including physical illness, QOL, medication adherence, number of friends, and calm coping mechanisms.

There is very limited research on the comorbidity of depressive symptoms in middle-aged and older adults with SP in China. Li et al. (9) conducted a study examining the prevalence, clinical correlates, and associated factors of MDE in first-episode and drug-naive patients with SP; however, this study primarily focused on young adults. Huang et al. (15) investigated suicidal tendencies and cognitive impairments in middle-aged and older adults with SP. In addition, the research by Xu et al. (16) mainly addressed the prevalence of depressive symptoms in Chinese adult male patients with SP. Despite various studies exploring the correlation between SP and MDE, there remains a significant lack of recognition regarding the clinical characteristics of SP combined with symptoms of depression in middle-aged and older adults in China. Studies are needed to identify depressive symptoms in patients with SP at an early stage by analyzing their clinical characteristics, enabling them to receive appropriate treatment promptly to improve their prognosis.

Therefore, this study aimed to investigate 1) the prevalence of depressive symptoms and clinical features of middle-aged and older adults with SP and 2) the clinical correlates and associated factors of depressive symptoms in this population.

## Materials and methods

### Participants

This study recruited participants admitted to the Department of Geriatric Psychiatry in a tertiary psychiatric hospital from February 2021 to March 2022 in Guangzhou (the capital city of Guangdong Province in China). The sampling site is the largest psychiatric specialist hospital in southern China, serving approximately 100 million community residents in Guangzhou and surrounding cities. The eligibility criteria for participation were as follows: 1) diagnosed with SP based on the International Classification of Diseases, 10th revision (ICD-10), the Diagnostic and Statistical Manual of Mental Disorders, 5th edition, or international diagnosis guidelines and 2) aged 40 years or older. The exclusion criteria were as follows: 1) comorbid severe physical or other psychiatric disorders, such as cardiovascular, neuromuscular, endocrine, or other somatic/substance use disorders, and 2) participants with severe hearing or visual impairment. Due to the particularity of the population, this study used convenience sampling to recruit participants. To mitigate potential bias, the sample size of this study was calculated using the prevalence estimation formula (Cochran 1977). The prevalence of SP combined with depression is generally over 50%, with a margin of error of 10% and a confidence level of 95% (*α* = 0.05); therefore, the minimum sample size is 96 participants. Considering a 15% non-response rate, it was ultimately necessary to recruit no less than 110 participants. The calculation formula is as follows:


$$n=\frac{Z^{2}\times P\times (1-P)}{d^{2}}$$

with *Z* = 1.96, *P* = 0.5, and *d* = 0.1.

### Procedures

The demographic characteristics and the clinical information, including age, gender, educational attainment, primary caregiver, marital status, first-time admission, alcohol use, smoking history, surgery history, psychiatric symptoms, modified electroconvulsive therapy (MECT) usage, physical restraints, and body mass index (BMI), were obtained from medical records. This study screened participants for depressive symptoms using the Geriatric Depression Scale (GDS), who were categorized into two groups: those with depressive symptoms and those without. A series of assessment tools were employed to evaluate the participants’ mental state, self-care ability, QOL, and social support. The Brief Psychiatric Rating Scale (BPRS) was used to assess the severity of the participants’ mental symptoms, while the Nurses’ Global Assessment of Suicide Risk (NGASR) was used to measure suicide risk. The Generalized Anxiety Disorder (GAD) scale was used to assess anxiety levels, and the Insight and Treatment Attitudes Questionnaire (ITAQ) was utilized to evaluate awareness of illness and the need for treatment. The Mini-Mental State Examination (MMSE) was used to assess cognitive function, the Schizophrenia Quality of Life Scale (SQLS) was used to measure QOL, the Social Support Rating Scale (SSRS) was used to evaluate social support, and the Barthel Index (BI) was used to assess the participants’ ability in activities of daily life (ADL). Variables that demonstrated statistical differences between the two groups were included as independent variables in the regression analysis to identify the risk factors for depression. All participants were required to complete assessments through interviews or observations conducted by two nursing graduate research assistants trained in the data collection procedures. Data collection was completed within 3 days of patient admission. This study received approval from the Ethics Committee of The Affiliated Brain Hospital, Guangzhou Medical University (approval no. 2022082). All patients provided written informed consent for participation, and their treatment adhered to the principles stated in the Declaration of Helsinki.

### Instruments

The GDS was created as a self-rating tool to assess depressive symptoms in older adults (17, 18). This study employed the 30-item version that captures both the affective symptoms (such as sadness, apathy, and crying) and cognitive domains (such as thoughts of hopelessness, helplessness, guilt, and worthlessness) associated with depression in the elderly. Using established cutoff points, the GDS scores can be interpreted as follows: scores of 0–10 indicate a normal range (no depression), 11–20 suggest mild depression, 21–25 indicate moderate depression, and 26–30 points indicate severe depression. The GDS was developed by Yesavage et al. (18), who assessed both normal elderly individuals and those taking antidepressants. Their findings demonstrated high reliability, with an internal consistency of 0.94, a split-half reliability of 0.94, a test–retest reliability of 0.85, and a criterion-related validity (in comparison to the Zung Self-Rating Depression Scale) of 0.84. In the study by Chan (19), the Chinese version of GDS was utilized to evaluate psychiatric elderly outpatients in Hong Kong, China. The results showed that this version maintained strong reliability, with an internal consistency reliability of 0.89, a test–retest reliability of 0.85, and a criterion-related validity (compared to psychiatrist diagnosis) of 0.95. In addition, the criterion-related validity (in comparison to the Center for Epidemiological Studies Depression Scale) was measured at 0.96 when using a cutoff score of >10 points. The sensitivity and specificity of the GDS were found to be 70.6% and 70.1%, respectively. The receiver operating characteristic (ROC) analysis exhibited strong classification accuracy, with an area under the curve (AUC) values of 0.92 for younger adults and 0.94 for older adults, and the sensitivity and specificity of the GDS for identifying depression were 72% and 97% for younger adults and were 86% and 91% for older adults, respectively (20). Consequently, this study set the cutoff point at 10, with patients scoring 10 or lower categorized as not exhibiting symptoms of depression, while those with scores exceeding 10 were considered to have depressive symptoms.

The BI is widely used to assess functional performance of ADL (21). The Chinese version of the BI has been extensively applied within older populations and has demonstrated high internal consistency and inter-rater reliability and a favorable concurrent validity. Scores on the BI range from 0 to 100, with 0–40 points indicating severe dependence, 41–60 points indicating moderate dependence, 61–99 points indicating slight dependence, and 100 points indicating complete independence.

The SQLS, which consists of 30 self-report items (each offering five response options: “never,” “rarely,” “sometimes,” “often,” and “always”), is used to assess the QOL of patients with SP across three domains: psychosocial (15 items), motivation/energy (seven items), and symptoms/side effects (eight items) (22). The scores for each domain range from 0 to 100, with higher scores indicating worse QOL. The total score of the SQLS is calculated by averaging the sums of the three domain scores.

The BPRS is used to assess the severity of psychotic symptoms (23). Each item is scored on a seven-point scale, where the scores correspond to the following levels of severity: 1) asymptomatic, 2) suspicious or very mild, 3) mild, 4) moderate, 5) mild severe, 6) severe, and 7) extremely severe. The BPRS is composed of five sub-dimensions, which include the following items: affect (anxiety, guilt, depression, and somatic); positive symptoms (thought content, conceptual disorganization, hallucinatory behavior, and grandiosity); negative symptoms (blunted affect, emotional withdrawal, and motor retardation); resistance (hostility, uncooperativeness, and suspiciousness); and activation (excitement, tension, and mannerisms–posturing).

The MMSE is the most widely used cognitive screening tool. It consists of 10 items, including orientation, registration, attention, calculation, recall, language tests, reading, and the ability to understand and obey commands. The total score on the MMSE ranges from 0 to 30 points, with a higher score indicating better cognitive function (24). The MMSE has demonstrated high sensitivity (87.6%-94.3%) and specificity (80.8%-94.3%) in the Chinese population the cognitive function of participants in this study (25).

The SSRS is used to measure individual social relationships (26). This scale consists of 10 items across three dimensions: subjective support (e.g., “How many intimate friends do you have? From whom can you receive support and help?”), objective support (e.g., “In the past, when you encountered difficulties, what was your source of comfort and caring?”), and the utilization of social support (e.g., “What is the way of talking when you are in trouble?”). The highest possible score is 66, with higher scores indicating greater social support. The scoring categories are as follows: scores below 22 indicate a low level of support, scores between 23 and 44 indicate a medium level of support, and scores from 45 to 66 indicate a high level of support.

The ITAQ was designed to assess awareness of illness and insight into the need for treatment in patients with SP (27). The questionnaire consists of 11 items scored on three 3-point Likert scales. Responses are scored from 0 to 2 points (0 indicates no insight, 1 indicates partial insight, and 2 indicates complete insight). The lowest score is 0 point, while the highest score is 22 points, with higher scores indicating greater insight.

The GAD explores how often individuals have experienced the seven core symptoms of generalized anxiety disorder over the last 2 weeks. Response options include “not at all,” “on some days,” “on more than half of the days,” and “almost every day.” Each response category is assigned a score of 0, 1, 2, or 3. The total score of the GAD ranges from 0 to 21, with score cutoff points at 5, 10, and 15 indicating mild, moderate, and severe anxiety levels, respectively (28).

The NGASR was designed to evaluate suicide risk-related predictors through 15 items. Previous studies have found significant correlations between certain variables and completed suicides, suggesting that not all indicators of suicide risk should carry the same weighting (29). In this scale, five items were assigned a “weighting” (or score) of 3, indicating a higher degree of suicide risk, while the remaining 10 items were assigned a “weighting” of 1. The maximum possible score of the scale is 25 points, with scores from 0 to 5 considered low risk, scores from 6 to 8 considered medium risk, scores from 9 to 11 considered high risk, and scores of 12 or higher considered severe high risk.

### Statistical analyses

Continuous data were expressed as the mean ± standard deviation (SD), and group comparisons were conducted using independent-samples *t*-tests. Categorical data were described using composition proportions. Categorical sociodemographic and clinical variables were analyzed using the chi-square test or Fisher’s exact test when 25% or more of the expected values in any cell of the R*C tables were below 5. Pairwise comparisons were performed with Bonferroni correction to minimize type I error. Spearman’s correlation was utilized to explore the relationship among the clinical characteristics, including the BPRS and its sub-dimensions, GAD, SSRS, ITAQ, NGASR, and SQLS. A correlation coefficient greater than 0.3 indicated a significant relationship between the two variables. A *p*-value <0.05 (two-tailed) was considered statistically significant. Logistic regression analysis was applied to identify potential predictors of depressive symptoms in patients with SP. For the regression analysis, standardized beta (*β*) coefficients with 95% confidence intervals (CIs) were calculated to assess the strength of the associations and their statistical significance.

## Results

### Demographic and clinical variables

A total of 116 patients were recruited between February 2021 and March 2022. **Table 1** illustrates the demographic and clinical characteristics of the participants. The mean age of the participants was 63.28 years (±7.87 years), with a range from 42 to 81 years. Among the 116 participants, 88 (75.9%) were women. There were no statistically significant differences in any of the demographic variables such as gender, age, education, marital status, smoking, alcohol use, first admission, surgery, physical restraints, and BMI between individuals with SP who exhibited depressive symptoms and those who did not.

**Table 1:** Demographic and clinical characteristics of middle-aged and older adults with schizophrenia (*N* = 116).

Variables	Categories	Total (*n* = 116)	Non-depression (*n* = 82)	Depression (*n* = 34)	*χ* ^2^/*t*	*p*
Gender	Men	28 (24.1)	21 (25.6)	7 (20.6)	0.029	0.865
	Women	88 (75.9)	61 (74.4)	27 (79.4)		
Age (years), *M* ± SD (min–max)		63.28 ± 7.87 (42–81)	62.91 ± 8.14 (42–81)	64.18 ± 7.18 (50–79)	−0.785	0.434
Education	Primary school and below	39 (33.6)	29 (35.4)	10 (29.4)	3.151	0.369
	Junior middle school	44 (37.9)	27 (32.9)	17 (50.0)		
	Senior middle school	24 (20.7)	19 (23.2)	5 (14.7)		
	University/College and above	9 (7.8)	7 (8.5)	2 (5.9)		
Marriage	Unmarried	13 (11.2)	11 (13.4)	2 (5.9)	1.749	0.417
	Married/remarried	81 (69.9)	57 (69.5)	24 (70.6)		
	Divorced/widow/widower	22 (18.9)	14 (17.1)	8 (23.5)		
Alcohol use	No	112 (96.6)	78 (95.1)	34 (95.1)	/	0.319
	Yes	4 (3.4)	4 (4.9)	0		
Smoking	No	112 (96.6)	79 (96.3)	33 (97.1)	/	1.000
	Yes	4 (3.4)	3 (3.7)	1 (2.9)		
First admission	Yes	80 (69.0)	56 (68.3)	24 (70.6)	0.059	0.808
	No	36 (31.0)	26 (31.7)	10 (29.4)		
Surgery (last 6 months)	No	115 (99.1)	82 (100)	33 (97.1)	/	0.293
	Yes	1 (0.9)	0	1 (2.9)		
Psychiatric symptom	Sensory impairment ^a^	1 (0.9)	0	1 (2.9)	/	0.293
	Thinking disorders	26 (22.4)	15 (18.3)	11 (32.4)	2.732	0.098
	Behavior disorder	70 (60.3)	49 (59.8)	21 (61.8)	0.041	0.840
	Emotional disorders	103 (88.8)	74 (90.2)	29 (85.3)	0.592	0.442
	Disturbance of intelligence	66 (56.9)	48 (58.5)	18 (52.9)	0.307	0.580
	Personality disorder ^a^	3 (2.6)	1 (1.2)	2 (5.9)	/	0.205
Physical restraints	No	107 (92.2)	73 (89.0)	34 (100)	/	0.057
	Yes	9 (7.8)	9(11.0)	0		
MECT	No	113 (97.4)	79 (96.3)	34 (100)	/	0.555
	Yes	3 (2.6)	3 (3.7)	0		
BMI, *M* ± SD (min–max)		21.72 ± 3.58 (14.22–29.78)	21.79 ± 3.49 (14.88–29.78)	21.55 ± 3.84 (14.22–27.69)	0.308	0.759

M, mean; SD, standard deviation; MECT, modified electroconvulsive therapy; BMI, body mass index.

^a^Fisher’s exact test.

### Comparison of the clinical characteristics between individuals with schizophrenia and depressive symptoms and those without

The percentage of individuals with SP who also manifested symptoms of depression was 29.31% (34/116). The results indicated statistically significant differences in various measures: the “affect” and “resistance” sub-dimensions of the BPRS and the GAD, ITAQ, NGASR, and SQLS scores between the two groups. As shown in **Table 2**, the “affect” sub-dimension of BPRS, along with the GAD, ITAQ, NGASR, and SQLS scores, was higher in the group with depressive symptoms compared with the group without depressive symptoms (all *p* < 0.05). Conversely, the “resistance” sub-dimension of BPRS was found to be lower in the group with depressive symptoms (*p* = 0.004).

**Table 2:** Comparison between patients with symptoms of depression and those without symptoms of depression in middle-aged and older adults (*N* = 116).

Variables		Total (*N* = 116)	Non-depression (*n* = 82)	Depression (*n* = 34)	*t*	*p*	95%CI
Barthel index, *M* ± SD (min–max)		88.88 ± 16.5 (20–100)	88.29 ± 17.2 (20–100)	90.29 ± 14.87 (45–100)	−0.593	0.555	
MMSE, *M* ± SD (min–max)		22.26 ± 6.82 (0–30)	22.01 ± 7.30 (0–30)	22.85 ± 5.58 (8–30)	−0.602	0.548	
BPRS		34.65 ± 7.59 (19–68)	34.48 ± 7.98 (19–68)	35.06 ± 6.65 (19–68)	−0.375	0.708	−3.66 to 2.50
Affect		6.29 ± 2.94 (0–17)	5.23 ± 1.67 (0–10)	8.85 ± 3.71 (4–17)	**−5.467**	**<0.001**	**−4.96** to **−2.28**
Negative symptoms		5.51 ± 2.81 (0–20)	5.42 ± 2.65 (0–20)	5.74 ± 3.19 (3–15)	−0.557	0.578	−0.15 to 0.82
Positive symptoms		9.92 ± 3.49 (4–18)	10.28 ± 3.37 (4–18)	9.06 ± 3.68 (4–16)	1.731	0.086	−0.17 to 2.62
Activation		4.42 ± 2.11 (0–13)	4.51 ± 2.22 (0–13)	4.20 ± 1.81 (1–9)	0.711	0.478	−0.55 to 1.16
Resistance		8.50 ± 3.17 (3–16)	9.04 ± 3.02 (3–15)	7.20 ± 3.18 (3–16)	**2.923**	**0.004**	**0.59** to **3.07**
GAD		5.45 ± 4.34 (0–18)	4.27 ± 3.29 (0–16)	8.29 ± 5.24 (0–10)	**−4.156**	**<0.001**	**−5.97** to **−2.07**
	None	54 (46.6)	44 (53.7)	10 (29.4)	**−3.322** ^a^	**0.001**	/
	Light	44 (37.9)	32 (39.0)	12 (35.3)			
	Moderate	12 (10.3)	5 (6.1)	7 (20.6)			
	Severe	6 (5.2)	1 (1.2)	5 (14.7)			
SSRS		27.72 ± 5.31 (16–42)	28.07 ± 5.49 (17–42)	26.85 ± 4.83 (16–36)	1.128	0.262	−0.92 to 3.36
ITAQ		3.86 ± 5.36 (0–22)	3.21 ± 5.13 (0–22)	5.44 ± 5.67 (0–19)	**−2.070**	**0.041**	**−4.37** to **−0.10**
NGASR		3.34 ± 2.83 (0–14)	2.62 ± 1.69 (0–9)	5.09 ± 4.08 (1–14)	**−3.407**	**0.002**	**−3.93** to **−1.00**
	Low	95 (81.9)	74 (90.2)	21 (61.8)	**−3.871**	**<0.001**	**/**
	Medium	11 (9.5)	7 (8.5)	4 (11.8)			
	High	7 (6.0)	1 (1.2)	6 (17.6)			
	Extremely high	3 (2.6)	0	3 (8.8)			
SQLS		32.51 ± 14.76 (9–87)	26.52 ± 9.77 (9–56)	46.94 ± 14.8 (23–87)	**−7.372**	**<0.001**	**−25.99** to **−14.84**

Bold values denote statistically significant *p* < 0.05 value.

*M*, mean; *SD*, standard deviation; *MMSE*, Mini-Mental State examination; *BPRS*, Brief Psychiatric Rating Scale; *GAD* Generalized Anxiety Disorder; *SSRS*, Social Support Rating Scale; *ITAQ*, Insight and Treatment Attitudes Questionnaire; *NGASR*, Nurses’ Global Assessment of Suicide Risk; *SQLS*, Schizophrenia Quality of Life Scale.

a Fisher’s exact test

### Correlations among GAD, ITAQ, SQLS, SSRS, NGASR, and GDS in people with schizophrenia

As shown in **Table 3**, the “affect” sub-dimension of BPRS was positively associated with the GDS score (*r* = 0.494, *p* < 0.01), the total GAD score (*r* = 0.359, *p* < 0.01), and the total SQLS score (*r* = 0.604, *p* < 0.01). In addition, the “affect” sub-dimension of the BPRS was positively associated with the GAD score (*r* = 0.470, *p* < 0.05) and the SQLS score (*r* = 0.577, *p* < 0.01). The GAD score was also positively correlated with the SQLS score (*r* = 0.400, *p* < 0.01). Furthermore, the NGASR score showed a positive association with the “affect” sub-dimension of the BPRS (*r* = 0.311, *p* < 0.01). Conversely, the ITAQ score was negatively associated with the “resistance” sub-dimension of the BPRS (*r* = −0.316, *p* < 0.01).

**Table 3:** Spearman’s correlation analysis.

Variables	BPRS	Affect	Negative symptoms	Positive symptoms	Activation	Resistance	GAD	SSRS	ITAQ	NGASR	SQLS
GAD	0.143	0.470*	−0.234*	−0.021*	0.217	0.008	–	–	–	–	–
SSRS	−0.188*	−0.018	−0.129	−0.080	0.108	−0.249**	−0.094	–	–	–	–
ITAQ	−0.200*	0.230*	−0.176	−0.178	0.026	−0.316**	0.289**	0.140	–	–	–
NGASR	0.182	0.311**	0.040	0.084	−0.132	0.015	0.225*	−0.192*	0.170	–	–
SQLS	0.225*	0.577**	0.061	−0.055	0.111	−0.118	0.400**	−0.045	0.177	0.286**	–
Depressive symptoms	0.024	0.494**	−0.001	−0.153	−0.043	−0.275**	0.359**	−0.086	0.213*	0.238*	0.604**

Bold values denote a correlation coefficient >0.3.

*p < 0.05 (two-tailed); **p < 0.01 (two-tailed).

BPRS, Brief Psychiatric Rating Scale; GAD Generalized Anxiety Disorder; SSRS, Social Support Rating Scale; ITAQ, Insight and Treatment Attitudes Questionnaires; NGASR, Nurses’ Global Assessment of Suicide Risk; SQLS, Schizophrenia Quality of Life Scale.

### Predictor of depressive symptoms in individuals with schizophrenia

The demographic and clinical characteristics were analyzed through logistic regression to identify predictors of depressive symptoms in individuals with SP. The results indicated that the "resistance" sub-dimension of the BPRS was inversely associated with the risk of depression (OR=0.790, 95%CI =0.648-0.963). The SQLS and GAD scores were significantly increased the odds of depression (OR=1.115, 95%CI=1.055-1.178, *p*<0.001 and OR = 1.205, 95%CI = 1.029-1.412, *p* <0.021, respectively) (see **Table 4**).

**Table 4:** Logistic regression analysis.

Independent variable	Assignment	OR	*p*	95% CI
Gender	Men: 0 Women: 1	/	0.565	/
Age	Value	/	0.429	/
Education	Primary school and below: 0 Junior middle school: 1 Senior middle school: 1 University/College and above: 1	/	0.678	/
Marriage	Unmarried: 0 Married/remarried: 1 Divorced/widow/widower: 1	/	0.207	/
Affect	Value	/	0.109	/
Resistance	Value	**0.790**	0.020	**0.648–0.963**
SQLS score	Value	**1.115**	**<0.001**	**1.055–1.178**
GAD score	Value	**1.205**	**0.021**	**1.029–1.412**
ITAQ score	Value	/	0.857	/
NGASR score	Value	/	0.447	/
Chi-square	75.528			
*R* ^2^	0.610			
*p*-value	<0.001			

Forward stepwise regression. Bold values denote a statistically significant *p* < 0.05 value (two-tailed).

GAD, Generalized Anxiety Disorder; ITAQ, Insight and Treatment Attitudes Questionnaire; NGASR, Nurses’ Global Assessment of Suicide Risk; SQLS, Schizophrenia Quality of Life Scale.

## Discussion

This study demonstrated the prevalence of depressive symptoms in patients with chronic SP. The clinical characteristics of middle-aged and older adults with SP who display depressive symptoms were compared with those who do not. Patients might exhibit both positive and negative symptoms of depression, but have not been diagnosed with MDE. These patients can be clinically classified as having symptoms of depression. Our main finding indicated that the “affect” sub-dimension of the BPRS and the QOL metrics were associated with depressive symptoms in middle-aged and older adults with SP.

A systematic review by Krynicki et al. (2018) suggested that the prevalence of depressive symptoms in individuals with SP ranges from 30% to 80%, depending on their ethnic background, the assessment tools used, the stage of the disease, and the treatment methods utilized (12). In this study, the incidence rates were slightly lower. Numerous studies found that depressive symptoms are significantly more common in patients with acute SP than in those with chronic SP. For example, a survey in the UK by Upthegrove et al. (2010) found that the prevalence of depressive symptoms was 60% during the acute phase and 50% in the chronic phase. Research by Siris et al. (5) indicated that the proportion of patients with depression in the USA ranges from 75% for first-episode SP to 7% for long-term hospitalized SP patients. After receiving formal antipsychotic treatment, the depressive symptoms of the hospitalized patients typically improved. Accordingly, future research could focus on first-time onset patients who have not yet received antipsychotic medication. Among middle-aged and older adults with SP, Zisook et al. (30) demonstrated that SDS was correlated with increases in the overall psychopathology, including both positive and negative symptoms, the severity of general medical conditions, impaired physical and mental functioning, and, possibly, more severe akathisia, alongside additional depressive symptoms. Our findings revealed that the “affect” sub-dimension of the BPRS, along with the GAD, ITAQ, and NGASR scores, was significantly higher in middle-aged and older adults with SP with depressive symptoms compared to those without. These results highlight that the affective symptoms, anxiety levels, and suicide risk are elevated in those with depressive symptoms, consistent with previous research (12, 31). Although this study found a low correlation between suicide risk and depressive symptoms in the logistic regression analysis, suicide risk was not a predictor of depressive symptoms in individuals with SP. This finding is in contrast with the study by Basu et al. (32), which identified suicidal intent as a strong predictor of depression in individuals with SP. This difference could have stemmed from our study participants, most of whom were receiving regular antipsychotic medication, whereas Basu et al. focused on untreated adults with first-episode SP. Consequently, while depressive symptoms might be prevalent among elderly patients with SP, clinical treatment would reduce the suicide risk.

Furthermore, insight into one’s illness could have both positive and negative impacts on those with SP. On the one hand, understanding their condition could improve compliance with treatment, particularly during the acute phase of mental illness (33). On the other hand, such an insight could worsen the feelings of low mood, disappointment, or depression and even increase the risk of suicide during the chronic or stable phases of the illness (34). As the majority of older adults are in the chronic phase of their condition, this might contribute to their depression. A higher insight into one’s illness has been identified as a risk factor for suicidality (35). Previous studies have illustrated the complex, nonlinear relationship between insight into illness and depressive symptoms in SP, influenced by factors such as social support (36), metacognitive ability (37), experiential avoidance (38), and internalized stigma (39).

Roseman et al. (40) discovered that insight and the severity of negative symptoms interact to impact QOL. This could be because individuals with SP experiencing depressive symptoms might hold a more negative outlook on life and underestimate their own QOL. Consequently, patients with SP experiencing depressive symptoms report lower QOL. The levels of anxiety and affect in patients with SP are correlated with their QOL. Our findings aligned with the conclusion of Zhou et al. (39) on the significant correlations between the total SQLS, PHQ-9, and GAD-7 scores, highlighting the notable influence of depressive and anxiety symptoms, as well as cognitive impairment, on the overall QOL. At the same time, our study did not show significant differences in the cognitive function between individuals with SP who exhibit symptoms of depression and those who do not. This result aligned with the findings of Zisook et al. (30). Majority of research studies focused on how depressive symptoms influence the QOL of patients with SP (41, 42). QOL has been used as an indicator to evaluate the outcomes of these individuals. However, the causal relationship between depression and QOL in individuals with SP remains unclear. It is uncertain whether SP leads to a decline in QOL, which then exacerbates depressive symptoms, or whether the decrease in QOL is a result of depressive symptoms. Our study indicated that QOL is a predictor of depressive symptoms in individuals with SP. This might be related to other factors that impact the participants’ QOL, subsequently influencing their level of depression. Currently, there are only a few studies demonstrating that a decline in QOL among patients with SP increases the risk of depression. More prospective longitudinal cohort studies are needed to examine the QOL of patients with SP before they develop symptoms of depression and explore other factors affecting QOL in order to better understand the causal relationship between QOL and depression.

Interestingly, the “resistance” sub-dimension of the BPSR emerged as a protective factor in the regression analysis. This suggests that, when patients with SP experience psychological symptoms such as hostility and suspicion, their emotions might become more easily stimulated than usual, which could, to some extent, mitigate depressive symptoms.

### Limitations

Data were collected from a single mental health hospital, which might limit the representativeness of the sample. In addition, the participants’ geographical area was restricted, and medication use was not taken into account. Therefore, the results should be interpreted with caution. Currently, there is limited research on GDS-30 in the younger and middle-aged populations, and a number of studies have utilized GDS-15, which is highly correlated with the GDS-30, in these demographics, demonstrating both high reliability and validity in older adults. Thus, the findings from this study regarding the use of GDS-30 in middle-aged individuals still hold clinical significance.

## Conclusion

In middle-aged and older adults with SP, those who experience symptoms of depression exhibit more affective symptoms, greater insight into their illness, higher levels of anxiety, and an increased risk of suicide. Among these individuals, affective symptoms and QOL are particularly associated with those who suffer from depression. Future research should focus on the relationship between the QOL of older adults with SP and the presence of depressive symptoms. In addition, exploring factors that contribute to the occurrence of depressive symptoms in older adults with SP would be important, and developing nursing programs aimed at reducing the incidence of depression would be necessary as well.

## Data Availability

The original contributions presented in the study are included in the article/supplementary material. Further inquiries can be directed to the corresponding authors.
